# Archaeological Artefact Database of Finland (AADA)

**DOI:** 10.1038/s41597-024-03602-8

**Published:** 2024-07-23

**Authors:** P. Pesonen, U. Nordfors, M. Roose, J. Saipio, J. Tiilikkala, U. Sanwal, V. Immonen, O. Vesakoski, P. Onkamo

**Affiliations:** 1 Department of Biology, University of Turku, Turku, Finland; 2 Department of Geosciences and Geography, University of Helsinki, Helsinki, Finland; 3 Archaeological Field Services, Finnish Heritage Agency, Helsinki, Finland; 4 Department of Archaeology, University of Turku, Turku, Finland; 5 Department of Geography and Geology, University of Turku, Turku, Finland; 6 Department of Archaeology, History, Cultural Studies and Religion, University of Bergen, Bergen, Norway; 7 Department of Finnish and Finno-Ugric languages, University of Turku, Turku, Finland

**Keywords:** History, Geography

## Abstract

This paper presents the Archaeological Artefact Database of Finland (AADA) of prehistoric (covering period of almost 11,000 years) artefacts in Finland that are categorised by type and are accompanied with photos of the artefacts. The database is intended to contain all typologically classifiable prehistoric artefacts found in Finland and held in Finnish collections. This dataset provides spatio-temporal context for artefacts across different time periods and regions, as it includes approximately 38,000 single artefacts and approximately 10,000 pottery type identifications from the Early Mesolithic to the end of the Iron Age in Finland (c. 8900 calBC - 1300/1500 calAD). In addition, the artefacts are given period-based (subperiod) dating to allow their chronological affiliation. To facilitate data usage, we also offer an R-script to replicate the data visualisation provided in this paper and a Python script to merge the artefact information to the pictures. We further work towards an interactive user interface for data download and visualization.

## Background & Summary

This paper presents the Archaeological Artefact Database of Finland (AADA), which includes comprehensive information on approximately 48,000 collection entries of Finnish archaeological artefacts and photos of these artefacts. The AADA dataset covers typologically classifiable tools and artefacts from the entire prehistory of Finland, from the beginning of pioneer settlement after the Last Ice Age (c. 8900 calBC) until the beginning of the medieval period (c. 1300 AD). Geographically, it covers the entire territory of present-day Finland, including the Åland Islands, and as well as artefacts collected before the Second World War from the territories ceded to Russia in 1945 (e.g., Karelia, Petsamo). The dataset will allow for varied statistical and modelling analyses, as well as the easy drawing of maps and site distributions.

Archaeological collections in Finland have been accumulated over the course of decades, first by the private collectors, then - along with the national awakening - by the historical societies and museums. Nowadays, the main keeper of the collections is the Finnish Heritage Agency, which takes care that both the heritage sites and artefact discoveries are tracked, registered, and protected by law^1^. A need for a digital archive arose from the tediousness of locating artefacts from various reports and scanned catalogues, but now AADA provides easy access to artefact locations, typology and phenotype. The closures of museums due to renovations and the pandemic of 2020–2021 further highlighted the need for an easy and costless entry to archaeological artefacts.

The creation of this archaeological dataset builds on an interdisciplinary collaboration involving numerous researchers and various fundings. The project began in 2009 and continued until 2023. The work has been preliminarily introduced by Nordfors *et al*.^2^.

The dataset is freely and openly available to anyone with the condition that this publication is mentioned as a reference when using the data in publications. By bringing together various resources into a single open data infrastructure, we promote FAIR principles^3,4^ for Finnish archaeological artefact data: Each artefact is now findable and accessible to a broad audience, eliminating the need to request artefacts from the museum archives. The systematic classification provides interoperability within the data as well as with new data that can be added to AADA. The coordinates also make the data interoperable with other spatially-explicit datasets. Moreover, the open access of AADA will promote reusability of the data and accelerate new types of analysis and visualisations while enhancing the longevity and sustainability of the dataset. To ensure maximal utilization, we are integrating AADA data into the interactive map platform Uralic Historical Atlas (URHIA) (https://sites.utu.fi/urhia/aada/)^5^, where users can generate data queries and create their own map visualisations of selected data. URHIA serves as a user-friendly platform for historical spatial datasets, with a particular emphasis on the geographical regions inhabited by speakers of Uralic languages, combining insights from archaeology and linguistics, especially focused on Finland.

**Fig. 1:**
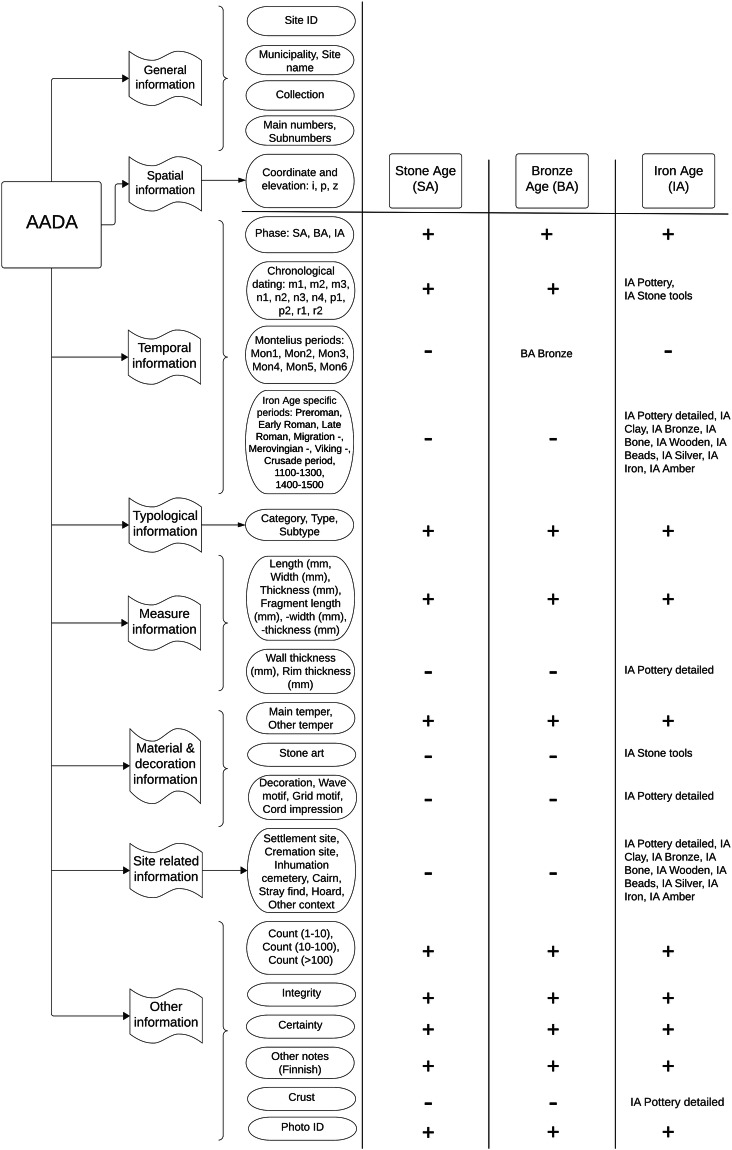
Structural overview of the Archaeological Artefact Database of Finland (AADA). Classification in AADA encompasses two overarching categories of attributes applicable across all periods: general and spatial information. Additionally, it incorporates six specific attribute types, namely temporal, typological, measure, material and decoration-related, site-related, and other attributes. For practical reasons, the data is divided into three distinct chronological periods: Stone Age (SA), Bronze Age (BA), and Iron Age (IA). + and - symbols denote whether or not certain attributes are available for certain periods. Attributes unique to specific artefact categories are identified accordingly. For instance, IA Pottery is elaborated with additional attributes specific to this category alone. These attributes include decoration (varied motifs) and measurement characteristics such as wall and rim thickness, as well as crust.

Our approach involves gathering information on-site from existing catalogues of museum collections, as well as conducting visual inspections and measurements of the artefacts in the collections. Other specific variable types collected were material and decoration-related, and site-related attributes (Fig. 1. We also included coordinates of the site where the artefact was found. The AADA dataset^6^, the related photos and a script to merge photos to the artefact information^7^, as well as the R-workflow producing the figures in this paper^8^ are deposited in Zenodo repository.

The AADA dataset covers a wide range of artefacts related to subsistence, social structures, cosmology, burial customs, and conflicts. It is important to note, however, that not all artefacts are equally well-represented in archaeological assemblages due to preservation issues, cultural practices, and recycling. Additionally, taphonomic processes, excavation practices, and the handling of finds have varied over time which affects the numbers and distributions of artefacts that have been preserved in museums and collections. Despite these limitations, existing archaeological collections provide an unparalleled source of geographically and chronologically attributable data for investigating changes and continuities in past human populations. The information on archaeological sites in Finland can be accessed through the *Kulttuuriympäristön palveluikkuna - Kyppi* (The Register of Archaeological Sites), which is maintained by the Finnish Heritage Agency.

Archaeological data is increasingly managed, analysed, and shared through digital platforms and analysed with computational tools and methods, such as GIS, digital recording systems, and statistical methods. AADA includes columns for latitude and longitude, transforming the data into a spatial point format that can be visualised and analysed using various software tools. Maps simplify complex data and provide a contextual view of multiple attributes, aiding in the interpretation and understanding of the data. To facilitate the creation of maps directly from the AADA dataset for those without prior expertise in working with spatial data, we provide an R-script that outlines the process used to create the maps presented in this publication. The AADA dataset offers a valuable resource for studying Finland’s prehistoric period and is accessible in Zenodo. The data will be continuously updated in the GitHub repository that will be managed by the Finnish Heritage Agency and University of Turku. New versions of AADA will be deposited to Zenodo in regular intervals^6^.

## Methods

### Creating the dataset

**Fig. 2:**
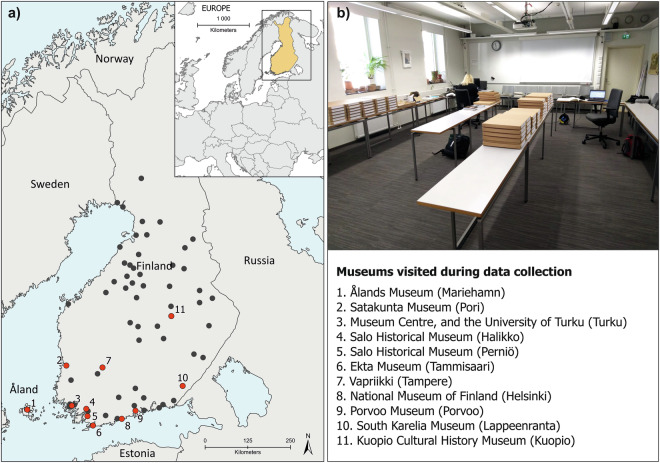
Overview of the data collection work in museums. Image (**a**) presents the distribution of the museums visited in 11 towns and image (**b**) presents the dataset creation process of manually inspecting the catalogues and collections, e.g., photographing the artefacts in museums. Black dots on the map represent minor museum collections that have not yet been included in the dataset. Background map: NaturalEarth.

The National Museum of Finland in Helsinki (Kansallismuseo, NM/KM) houses the majority of Finnish archaeological collections, with approximately 90% of all archaeological material discovered in Finland deposited there. Other important collections are located in Mariehamn (Ålands Museum, ÅM), Pori (Satakunta Museum, SatM), Turku (Turku Museum Centre, TMM; University of Turku, TYA), Tampere (Former Häme Museum, HM, in Museum Centre Vapriikki), Kuopio (Kuopio Cultural History Museum, KHMESIE), and Oulu (Northern Ostrobothnia Museum, PPM). We studied all these major collections within our project, but due to the lack of resources and pandemic closures we had to leave Oulu out. In addition to these, some smaller county museums were also inspected (Fig. 2).

Each entry in the catalogue of the original collections may contain 1–20,000 separate artefact entries, e.g., excavation of a site may include altogether 10,000 single items, which are organised in some hundreds of subnumbers. The majority of the single items are so-called massfinds, e.g., stone debitage, bones, and pottery sherds, and only a minority are typological artefacts. Still, the AADA dataset has records of over 37,000 typological artefacts and c. 10,000 pottery type definitions (Table 1).

**Table 1:** The count of items in the AADA dataset (version 1) by item category in three time periods: Stone Age (c. 8900–1900 calBC), Bronze Age (c. 1900–500 calBC), and Iron Age (c. 500 calBC–1300 calAD).

Category	Stone Age	Bronze Age	Iron Age	AADA total
Pottery	7,343	861	1,668	9,872
Pottery (detailed)	0	0	822	822
Stone artefacts	29,126	329	334	29,789
Clay artefacts	1,159	15	195	1,369
Bone artefacts	567	72	98	737
Wooden artefacts	397	2	1	400
Amber artefacts	1,016	0	1	1,017
Birch bark tar	383	28	0	411
Silver and golden artefacts	0	0	83	83
Bronze artefacts	0	167	877	1,044
Iron artefacts	0	0	1,399	1,399
Beads	0	0	992	992
Total	39991	1474	6470	47935

In the pottery category, the figures indicate the presence of certain pottery types, while in other categories, the figures represent the actual number of items.

### Temporal information

For pragmatic reasons, the dataset has been divided into three main chronological periods: Stone Age, Bronze Age, and Iron Age. AADA applies the currently used periodization of prehistory in Finland (Table 2). Each period contains both period-specific and general artefact categories (Table 1) and accordingly also period-specific and general attributes (Fig. 1). The general item categories are pottery, stone tools, bone tools, clay artefacts, and wooden artefacts. Period-specific categories include amber implements specific to Stone Age, and birch bark pitch occurrences to Stone and Bronze Age. Bronze objects are specific to Bronze and Iron Age. The Iron Age includes separate tables for iron artefacts, beads made of different materials, and silver and gold items (including a single Bronze Age gold item). Gold artefacts are extremely rare in Finland in the Iron Age, but bronze objects may have been gilded. These specific artefacts are included in the bronze category. Coins, which have occasionally been used as pendants, are included in the silver find category. In all categories, the presence of organic remains, such as textile, fur or wood fragments, was documented.

**Table 2:** The AADA dataset applies the prehistorical periodisation of Finland, divided more specifically as periods, subperiods, calibrated years before Christ (calBC/AD), and calibrated years before present (BP).

Phase	Period	Subperiod	calBC/AD	calBP
Stone Age	Mesolithic	Early Mesolithic (m1)	8900–8200 BC	10900–10200
		Middle Mesolithic (m2)	8200–6200 BC	10200–8200
		Late Mesolithic (m3)	6200–5100 BC	8200–7100
	Neolithic	Early Neolithic (n1)	5100–3900 BC	7100–5900
		Middle Neolithic (n2)	3900–2900 BC	5900–4900
		Late Neolithic (n3)	2900–2400 BC	4900–4400
		Final Neolithic (n4)	2400–1700 BC	4400–3700
Bronze Age	Early Bronze Age (p1)	Montelian period I (Mon I)	1700–1500 BC	3700–3500
		Montelian period II (Mon II)	1500–1330 BC	3500–3330
		Montelian period III (Mon III)	1330–1100 BC	3330–3100
	Late Bronze Age (p2)	Montelian period IV (Mon IV)	1100–950 BC	3100–2950
		Montelian period V (Mon V)	950–800 BC	2950–2800
		Montelian period VI (Mon VI)	800–500 BC	2800–2500
Iron Age	Early Iron Age (r1)	Pre-Roman Iron Age	500–1 BC	2500–2000
		Early Roman Iron Age	1–200 AD	2000–1800
		Late Roman Iron Age	200–400 AD	1800–1600
		Migration Period	400–600 AD	1600–1400
	Late Iron Age (r2)	Merovingian Period	600–800 AD	1400–1200
		Viking Age	800–1050 AD	1200–950
		Crusade Period (Early Medieval Period)	1050–1250	950–750
		Medieval Period	1250–1530	750–470

### Typological information

The principles of recording the artefacts into the dataset were kept as constant as possible throughout the project. The scope of the dataset was limited to typologically discernible tools and artefacts. In the case of stone tools, a particular emphasis was placed on polished items, given their generally clearer recognition and classification attributes. Recording of chipped stone tools encompassed primarily surface-retouched arrow/spear points, daggers, sickles, and axes, all of which adhere to established typological criteria. A variety of informal small tools, such as scrapers and knives, were thus left out of the dataset. While these tools would offer information through their distribution and possible provenance, so far they lack a concise typology. Moreover, scrapers are so numerous that adding them into the dataset would have been too time consuming.

In terms of pottery finds, recognizable types and the relative quantities of sherds associated with them were covered under each catalogue main number., i.e., it was not attempted to separate single vessels from the material, the exception being – in some cases – Iron Age pottery. Within the Iron Age material, certain bulk finds (clay daub) and objects that are difficult to identify to a specific sub-period (knives, rings), were not recorded as they generally do not significantly contribute to a dataset concentrating on chronologically and typologically identifiable objects. It should be noted that many of the Iron Age finds in Finland originate from sites that may have been used for centuries, and the find contexts do not always provide a clear indication for exact dating of the material; some of the items, such as certain iron tools and ceramic types, were used until the Middle Ages.

Apart from the measurable qualities of the artefacts, the information gathered is subjective to some degree. Most notably, type definitions and in some cases also mineralogical identifications of material are relatively intuitive and should be treated as such. We limited the number of people filling in the dataset to ensure as much consistency in typological interpretation as possible: Stone Age entries were made only by P. Pesonen and J. Saipio, Bronze Age entries solely by J. Saipio, and Iron Age entries by U. Nordfors and J. Tiilikkala. Relevant source publications were referenced periodically. The consistency of identifications will help future work if categorizations have to be modified or corrected. During the collection work, almost all the items were also photographed so as to include them as part of the dataset^7^.

### Spatial information

**Fig. 3:**
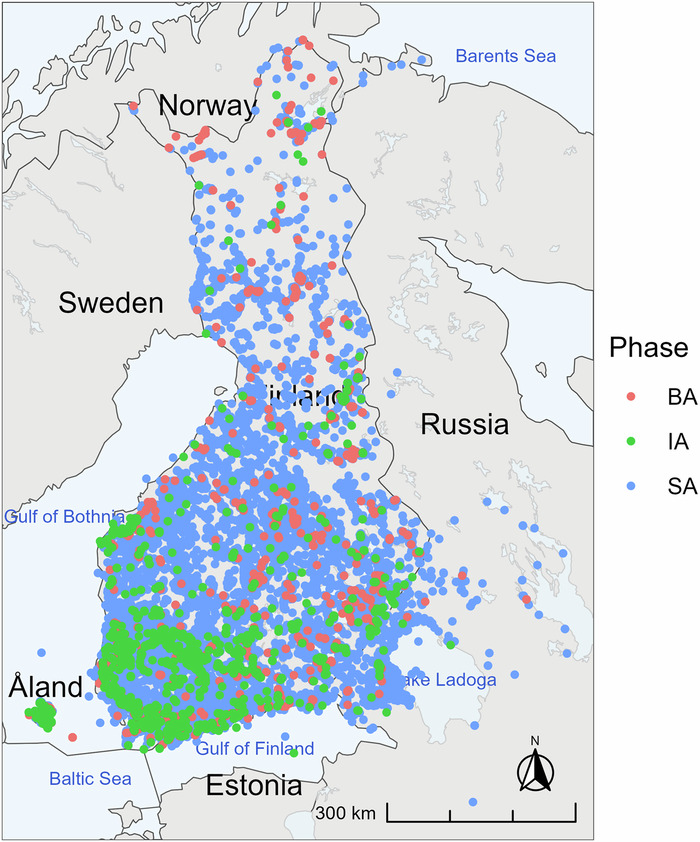
The geographical distribution of items (n = 47935) in the AADA dataset, divided into three chronological periods (phases): Stone Age (SA), Bronze Age (BA), Iron Age (IA).

AADA records the geographical context of artefacts, detailing where each was discovered from. Unlike characteristics which are period-specific and vary based on the era being studied, spatial information has a universal relevance across all time periods. It provides a foundational framework for understanding the geographical distribution, relationships, and contextual significance of the artefacts and archaeological findings. The dataset predominantly focuses on Finland (see Fig. 3). It contains geographical coordinates for each artefact, facilitating the creation of maps that visually represent find distributions clearly and in an easily accessible manner. Importantly, these maps enable the assessment of spatiotemporal relationships and trends between artefact and material types. AADA has already been utilised for showcasing this in earlier phases of the dataset project^9–13^.

## Data Records

The AADA dataset is available at Zenodo^6–8^ and future updates will be done in GitHub.

The AADA dataset is thematically organised into three Excel workbooks according to the chronological periods: Stone Age, Bronze Age, and Iron Age (Table 1). Each row in the dataset represents a single artefact, while each column represents a specific attribute, such as the type, period, site, and measurements of the artefact. Moreover, each workbook contains spreadsheets based on the artefact type (Fig. 1). For example, for the Stone Age, there are separate tables for pottery, stone tools, clay artefacts, bone artefacts, wooden artefacts, amber, and birch bark tar. Similarly, for the Bronze Age, there are separate tables for pottery, stone tools, clay artefacts, bone artefacts, wooden artefacts, and bronze objects. For the Iron Age, there are separate tables for pottery, stone tools, clay artefacts, bone artefacts, wooden artefacts, bronze artefacts, iron artefacts, silver and golden artefacts, beads, and organic materials. In addition, due to the chronological period-dependent recording procedures, the Iron Age table also contains a detailed table of pottery and stone tools (where the dating is specified according to the Iron Age subperiods).

All other tables, except the ones about pottery, are constructed by having an artefact in each row. Within the pottery table, each row denotes the presence of a certain pottery type in a certain collection number, i.e., the presence does not record how many vessels etc. there are on the collection number. Some measure of the quantity is expressed in “count” columns (see below).

### General and spatial information

The AADA dataset contains primary information on archaeological artefacts from different prominent institutions across Finland, such as the National Museum of Finland, Ålands Museum, and Turku Museum Centre, amongst others. These artefacts are organised using a main number and sub-number system. Additionally, the dataset includes information on the municipality. ***Collection*** contains source information of 32 museums: KM = Kansallismuseo (National Museum of Finland); ÅM = Ålands Museum; TYA = Turun Yliopisto Arkeologia (University of Turku, Archaeology); TMM = Turun museokeskus (Turku Museum Centre; current signum is TMK but dataset uses TMM); SatM = Satakunnan Museo (Museum of Satakunta); KHMESIE = Kuopion kulttuurihistoriallinen museo (Kuopio Cultural History Museum); EKM = Etelä-Karjalan museo (Museum of South Karelia); BM = Porvoon Museo (Porvoo Museum, sw. Borgå Museum); HM = Hämeen museo (Former Häme Museum, in Museum Centre Vapriikki, Tampere); Hal = Halikon museo (Halikko Museum, part of the Salo Historical Museum); Per = Perniön museo (Perniö Museum, part of the Salo Historical Museum); Linder = Linder collections in Turku Museum Centre; Nyberg = Nyberg collections in EKTA Museum Raasepori; SII = Pöljän kotiseutumuseo (curated by Kuopio Cultural History Museum); KARTT/VI = Karttulan kotiseutumuseo (curated by Kuopio Cultural History Museum); KIUR = Kiuruveden museo (Kiuruvesi Museum, curated by Kuopio Cultural History Museum); Lauri Nautela kok = Lauri Nautela Museum, Lieto; SHH = Stockholm Historiska Museet, Sweden.

#### Main numbers and Sub-numbers

organise collections, e.g., KM 12456:1–25, where KM indicates the National Museum of Finland’s collection with the main number 12456, which has sub-numbers 1 to 25.

#### Municipality, Site id and Site name

The municipality categorises archaeological artefacts by their place of origin on a municipality-wise basis. The dataset reflects the situation as of 2020, although several municipalities have since merged. Old parish names are used in the regions ceded to the Soviet Union (names prior to 1945). Site names and identification numbers are in accordance with the Register of Archaeological Sites curated by the Finnish Heritage Agency.

#### Coordinates (p/i/z)

AADA’s coordinate reference system (CRS) is World Geodetic System (version WGS 84) where x stands for northing and y stands for easting. The dataset was originally compiled using “KKJ / Finland Uniform Coordinate System”, with the EPSG identifier 2393 (https://epsg.io/2393). Z is for the elevation (meters) of the site above sea level, “i” stands for easting (“itä” in Finnish), and “p” stands for northing (“pohjoinen” in Finnish”). KKJ reference system is also available.

### Temporal information

#### Phase

Information on the chronological period of the artefact, with SA denoting the Stone Age (8900–1900 calBC), BA representing the Bronze Age (1900–500 calBC), and IA indicating the Iron Age (500 calBC–1300 calAD).

#### Period/dating

The dating of the artefacts in the AADA dataset varies according to material. In most tables (pottery, stone tools, clay, bronze, iron, beads) the dating is based on the typology of the artefacts. In some tables, other datable finds from the same site are used as dating criteria, i.e., thus providing a wider range of dating options for the object, including materials such as amber, bone, birch bark, and wood. The periodization for the Late Iron Age differs in western, eastern, and northern Finland. The Late Iron Age is generally extended to at least c. 1300 AD in Eastern Finland and Karelia, and occasionally even longer in Northern Finland.

### Typological information

#### Typology (category, types, subtypes)

The artefacts in the dataset are organised hierarchically by typology, which includes category, type, and subtype. For example, the category of Stone Age bark floats is “wooden artefact,” the type is “fishing implements,” and the subtype is “bark float.” In some cases, such as Stone Age stone tools, there is also a lower subtype hierarchy (subtype 2). In addition, Finnish terms for subtype (and subtype 2) are presented as well.

### Site context information

#### Settlement, cremation cemetery, inhumation cemetery, cairn, stray find, hoard, other context

For Iron Age artefacts, the main type of the find context is also recorded: inhumation cemetery, cremation cemetery, cairn, settlement site, hoard, or stray find. This attribute gives contextual information on the find circumstances, which is crucial for understanding the distribution of the Iron Age artefacts.

### Other information

#### Object attributes (certainty, integrity, measures, crust, etc.)

Object attributes, such as integrity and measures, differ depending on the material. Certainty indicates the dataset compiler’s subjective identification of the artefact type, with 1 meaning certain, 2 meaning probable, and 3 meaning possible. The integrity is indicated with a TRUE/FALSE statement in the relevant column. The intact and fragmented artefacts’ dimensions are recorded in separate columns (length, width, thickness). For pottery, the main temper and other tempers are explained in two columns, currently in Finnish. If present, the decorative motifs on Iron Age pottery are recorded. However, the documentation of stone tool material is only available in Finnish and is based on a quick and superficial visual inspection, making it very subjective. Therefore, the recorded information on stone tools should only be considered suggestive. For Iron Age pottery, a separate “crust” column was used to record the presence of food crusts in the surfaces of the pottery. In Stone and Bronze Age pottery this is written (in Finnish, “karsta”) in the other notes column.

#### Count (only pottery)

The number of pottery sherds is recorded in three columns (count 1–10, count 10–100, and count v>100 sherds) to indicate their relative amount.

#### Other notes

This column has some additional information in Finnish. The essential typological information is translated to English. We also include the non-translated, within-team comments to the data for they could be of interest to Finnish archaeologists. Translations of these will be added in the next version.

#### Photos

Each photo has an individual name (ID) and the individual photo is found in the corresponding folder. Most of the pottery data do not have photos of them because the material is highly fragmented and unique vessels were not separated. Photo names follow the pattern “Collection” + “Main number” + “Subnumber” (e.g., KM 12345_5.jpg). Multiple photos of the same artefact are distinguished by appending letters *a, b, c* etc., (e.g., KM 12345_5b.jpg). Users can easily locate photos by navigating the correct folder. Photolinks can also be created to the Photo ID column of the Excel tables. Users need to download the photos to their computer and run the Python script available in the AADA Photos Hyperlink folder. The details of the script can be found in the Readme file of the same folder.

## Technical Validation

The artefacts kept in the collections of the National Museum of Finland, regional museums and various local museums, form the backbone of the archaeological record in Finland. The ancient sites and artefacts have been protected by law and they have been the property of the government since the 1600 s. There is no particular geographical or typological skew in museum collections across Finland. However, distortions may exist due to modern land use patterns dictating where archaeological rescue excavations have taken place. The geographical locations of the find spots are in most cases confirmed by professional archaeologists, and nowadays with the help of GPS instruments. It is worth noting that the locations of some of the oldest finds may only indicate the parish, village, or farm where they were discovered. This should not, however, be a major disadvantage.

## Usage Notes

The archaeological artefact dataset forms the baseline for any artefact-oriented study in Finland. Moreover, its relevance extends to the prehistory of neighbouring regions, including Northwest Russia, Estonia, the Baltic area, Scandinavia and Sápmi.

Certain aspects still require attention, particularly the completion of data regarding Iron Age artefacts. Approximately 45–50% of the Finnish Iron Age material needs to be added to the dataset (excluding the metal detector finds made during the past two decades)^1^. Additionally, the documentation of Stone Age stone tools is not entirely complete. Many local museums in Finland possess a few to a few dozen stone axes and chisels, which were not possible to record during the current project. Within the collections of the National Museum of Finland, recording of stone artefacts acquired in the 1910s and 1920s is also incomplete. Similarly, the collections of Ålands Museum and Tampere Museum also lack comprehensive documentation. Presently, the geographical focus of the dataset emphasises Southern Finland since it was not possible to study the collections of museums in Northern and Eastern Finland. This was due to the lockdown of museums during the COVID-19 pandemic in 2020.

The integration of geospatial information into the dataset provides a valuable resource for digital humanities studies. This involves exploring the interaction between spatiality and temporality, while considering aspects such as scalability and representativeness. However, the primary advantage of the dataset lies in its potential to advance digital humanities research.

### Disclaimer

This Data Descriptor was peer-reviewed in 2024 using the data available on the platform at that time. Dataset may undergo updates and changes over time. For the most current information, refer to the latest version available on the platform.

**Fig. 4:**
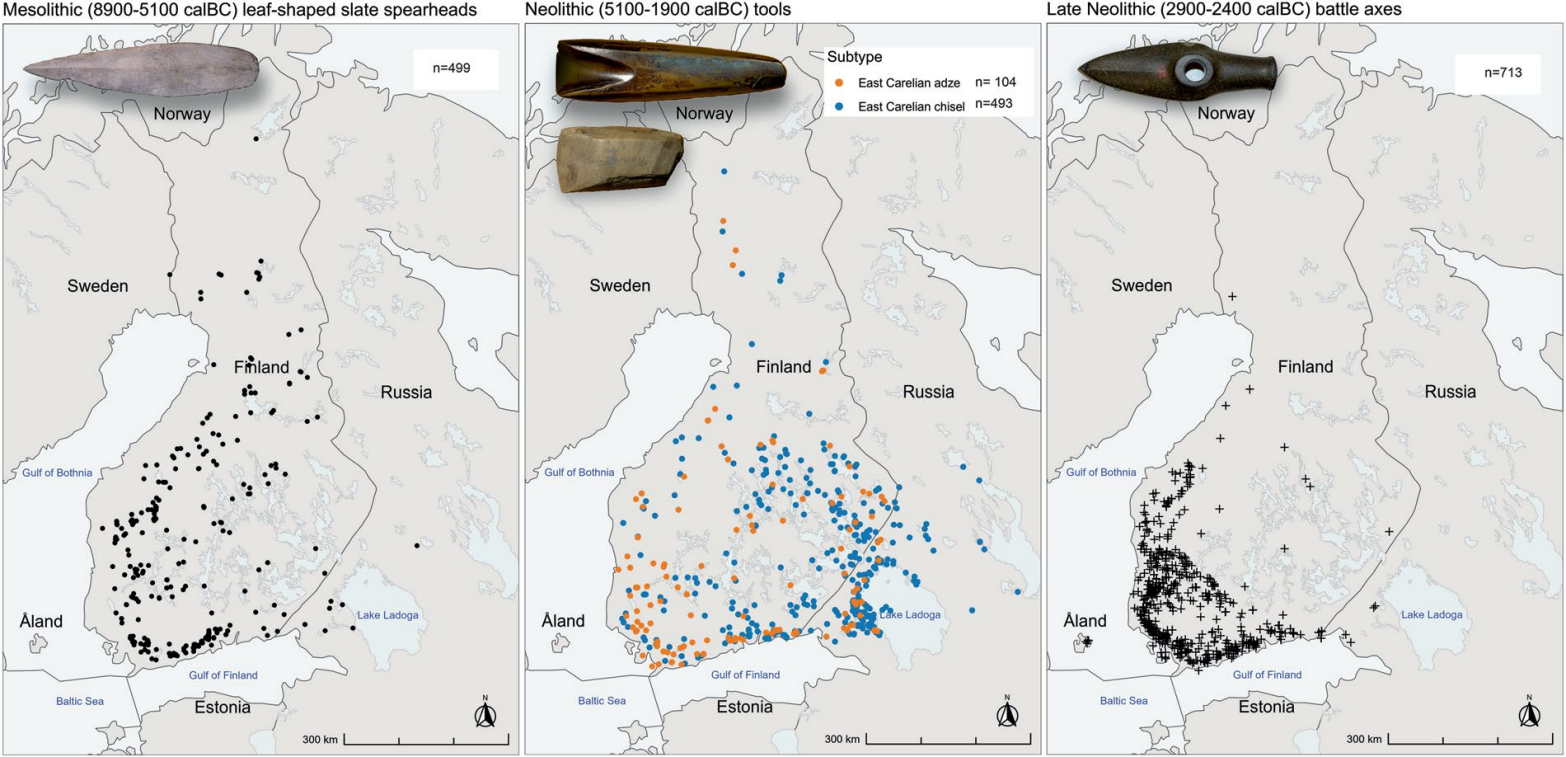
Examples of Stone Age stone tool entries plotted on the map of Finland. (**a**) Leaf-shaped slate spearheads from the Mesolithic period (spearhead from Jalasjärvi Laulaja, KM 20648:2, (**b**) East Carelian adzes and chisels from Early and Middle Neolithic periods (adze from Parikkala, KM 253, chisel from Räisälä Kökkölä, KM 1922:232), and (**c**) Battle axes from the Late Neolithic period (battle axe from Sastamala Tyrvää, KM 452). Photos by Petro Pesonen.

**Fig. 5:**
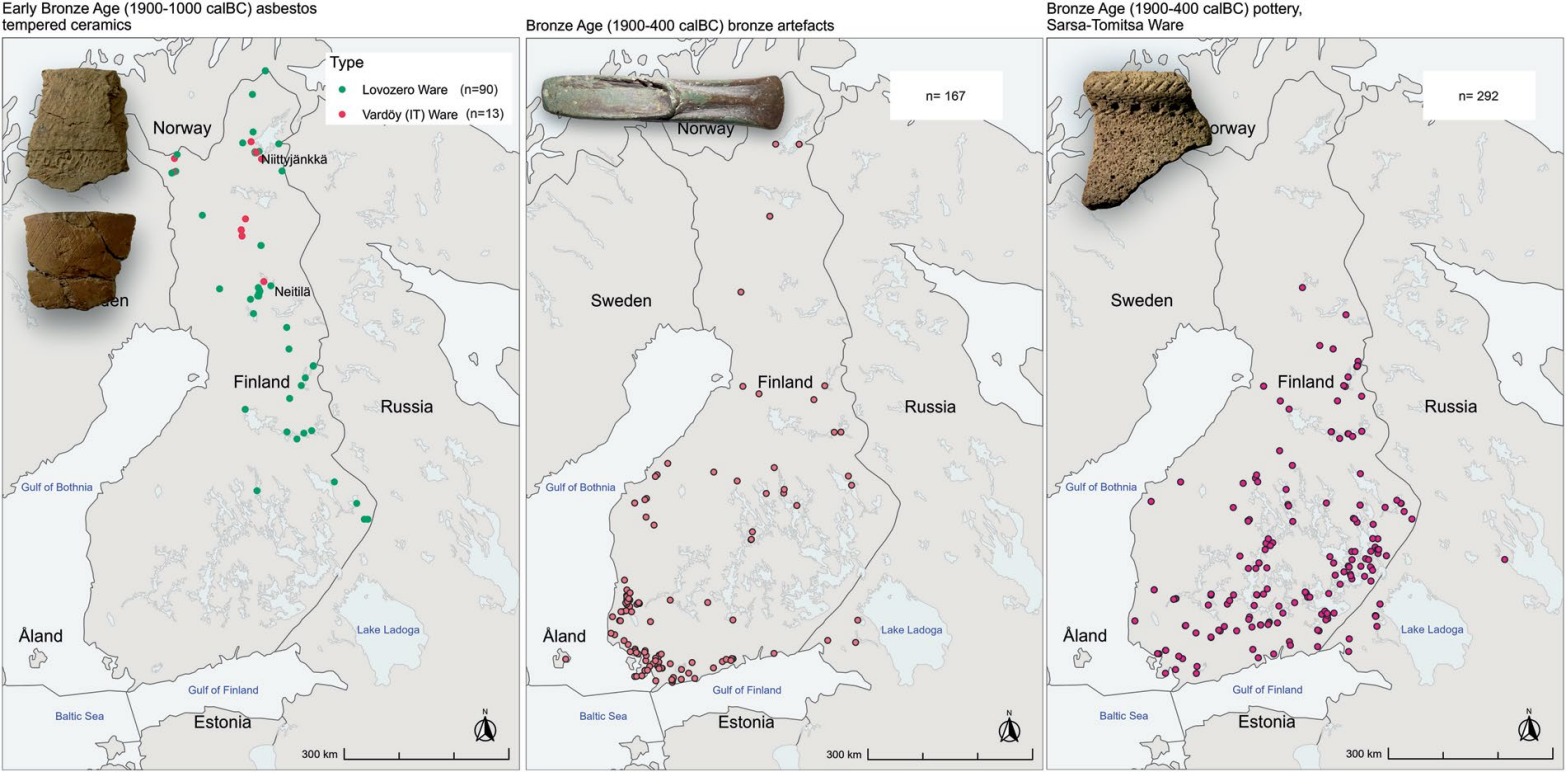
Examples of Bronze Age artefacts (c. 1900–500 calBC) plotted on the map of Finland, (**a**) Early Bronze Age pottery of Northern Finland: Lovozero Ware (green dots, the sherd in left from Kemijärvi Neitilä 4, KM 16145:2122, photo Petro Pesonen) and Vardöy Ware (also called Imitated Textile (IT) pottery, red dots, the sherd in right from Inari Niittyjänkkä, KM 26240:1, photo Petro Pesonen), (**b**) Bronze Age artefacts of bronze (palstave, KM 14532 from Raasepori Landsbroström, photo Jarkko Saipio), (**c**) Sarsa-Tomitsa Ware (KM 22495:1 from Virolahti Mattila, photo Petro Pesonen).

**Fig. 6:**
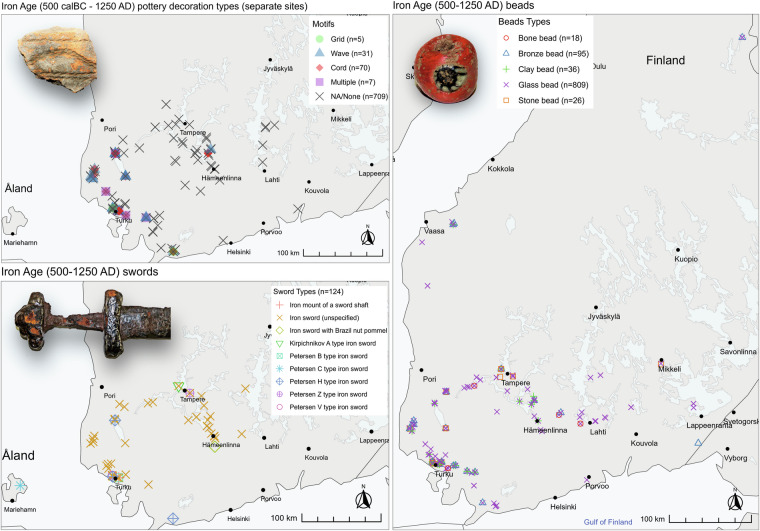
Examples of Iron Age artefacts plotted on the map of Finland, (**a**) Iron Age pottery decoration types (wave decoration on a sherd from Raasepori Domargård, KM 21634:1648), (**b**) Iron Age sword types (a sword from Eura Luistari, KM 17847:1), (**c**) Iron Age bead types (a glass bead from Lahti Paakkolanmäki, KM 21967:1). All photos by Ulla Nordfors.

**Fig. 7:**
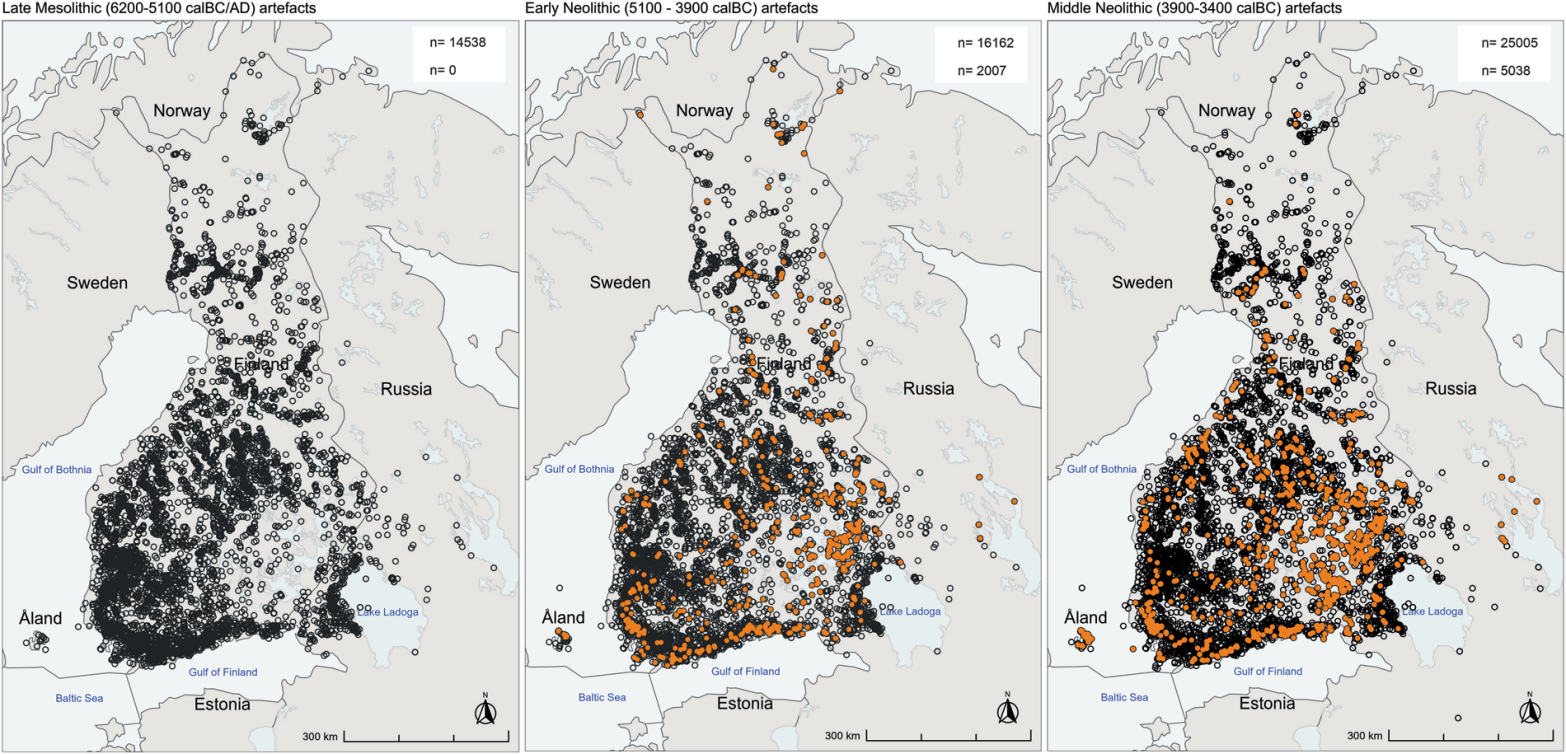
Examples of period-wise plotted artefact distributions, (**a**) Late Mesolithic artefacts (black dots, c. 6200–5100 calBC), (**b**) Early Neolithic artefacts (c. 5100–3900 calBC, orange dots (n = 2007) denoting Early Neolithic pottery and clay artefacts; black dots (n = 16162) all the other Early Neolithic artefacts), and (**c**) Middle Neolithic artefacts (c. 3900–3400 calBC, orange dots (n = 5038) denoting Middle Neolithic pottery, clay, and amber artefacts; black dots (n = 25005) all other Middle Neolithic artefacts). The intensification of the “Neolithic” type artefacts (pottery, clay artefacts and amber) can be observed throughout Southern Finland and especially in the Southeastern Lake Saimaa region, which was transgressive until the beginning of the Middle Neolithic, 3900 calBC^14^.

## Data Availability

No custom code was used in the collection and creation of the data. Scandinavian letters (like “å”, “ä”, and “ö” in Swedish and Finnish) are utilized in place names and descriptive sections. A Python code for merging photographs with artefacts is provided alongside the AADA photos in the Zenodo repository (Supplement 2)^7^. The R-markdown document and tailored R-script (.rmd) provided allow for the replication of spatial distributions of stone tool typologies during the Stone Age (Fig. 4), variations in artefacts during the Bronze Age (Fig. 5) and Iron Age (Fig. 6), and changes in overall activity from the Late Mesolithic to the Middle Neolithic (Fig. 7). Users have the capability to selectively query relevant portions of the dataset, facilitating accelerated data exploration and visualization processes (Supplement 3)^8^.
